# Evaluation of the Performance of Five Diagnostic Tests for *Fasciola hepatica* Infection in Naturally Infected Cattle Using a Bayesian No Gold Standard Approach

**DOI:** 10.1371/journal.pone.0161621

**Published:** 2016-08-26

**Authors:** Stella Mazeri, Neil Sargison, Robert F. Kelly, Barend M. deC. Bronsvoort, Ian Handel

**Affiliations:** 1 The Roslin Institute, University of Edinburgh, Edinburgh, United Kingdom; 2 Royal (Dick) School of Veterinary Studies, University of Edinburgh, Edinburgh, United Kingdom; 3 Farm Animal Clinical Sciences, School of Veterinary Medicine, University of Glasgow, Glasgow, United Kingdom; University of Texas Medical Branch, UNITED STATES

## Abstract

The clinical and economic importance of fasciolosis has been recognised for centuries, yet diagnostic tests available for cattle are far from perfect. Test evaluation has mainly been carried out using gold standard approaches or under experimental settings, the limitations of which are well known. In this study, a Bayesian no gold standard approach was used to estimate the diagnostic sensitivity and specificity of five tests for fasciolosis in cattle. These included detailed liver necropsy including gall bladder egg count, faecal egg counting, a commercially available copro-antigen ELISA, an in-house serum excretory/secretory antibody ELISA and routine abattoir liver inspection. In total 619 cattle slaughtered at one of Scotland’s biggest abattoirs were sampled, during three sampling periods spanning summer 2013, winter 2014 and autumn 2014. Test sensitivities and specificities were estimated using an extension of the Hui Walter no gold standard model, where estimates were allowed to vary between seasons if tests were a priori believed to perform differently for any reason. The results of this analysis provide novel information on the performance of these tests in a naturally infected cattle population and at different times of the year where different levels of acute or chronic infection are expected. Accurate estimates of sensitivity and specificity will allow for routine abattoir liver inspection to be used as a tool for monitoring the epidemiology of *F. hepatica* as well as evaluating herd health planning. Furthermore, the results provide evidence to suggest that the copro-antigen ELISA does not cross-react with *Calicophoron daubneyi* rumen fluke parasites, while the serum antibody ELISA does.

## Introduction

Fasciolosis, first reported in 1379, has been recognised as a clinically and economically important disease for centuries [1]. The infection caused by trematode parasites of the genus *Fasciola* can infect many mammals including sheep, cattle, goats, deer and humans [2]. In cattle, fasciolosis mainly manifests in its chronic form, which can lead to weight loss, anaemia and hypoproteinaemia. Clinical signs are often mild and may present as loss of productivity, while in severe cases sub-mandibular oedema may be seen. Unlike sheep, cattle liver pathology includes bile duct calcification and gallbladder enlargement [2, 3]. Globally, the infection is estimated to cost the livestock industry €2.5 billion per year [4], while losses due to liver fluke have been estimated to range between €1100-2000 million per year in the European Union [5].

In the UK and other temperate regions, *F. hepatica* is the most common aetiological agent of fasciolosis [2]. *F. hepatica* has a complicated multi-host, highly climate dependent life cycle which takes typically between 18 and 30 weeks to be completed. The mud snail, *Galba truncatula* is the most common intermediate host of *F. hepatica* in Europe [3, 6]. Temperature and moisture levels play an important role in the parasite’s life cycle and it is generally accepted that average daily temperatures of more than 10°C and high moisture levels are required for both the egg development and the reproduction of the parasite within the snail [7]. This results in seasonal increases of the incidence of infection, which vary between years depending heavily on climatic conditions.

The incidence of fasciolosis in the UK has been reported to have increased during the last decade and more importantly its distribution has changed. In the past, fasciolosis was most commonly seen in the wetter western regions of the country, while it is now evident that the disease has become endemic in the previously drier eastern regions [8, 9]. Reasons for the changing epidemiology of *F. hepatica* are thought to include climate change, increasing animal movements and development of triclabendazole resistance [10]. Unpredictable weather conditions and resistance to anthelmintic treatment make control strategies less straightforward to plan. This increases the need for appropriate use of diagnostic tests, which along with improved knowledge and consideration of their limitations, can enhance implementation of more effective management strategies.

The development of tests for the correct diagnosis of the infection has been going on for years, yet no test developed so far has been shown to have adequately high sensitivity and specificity in the field setting. Research on performance of available diagnostic tests in cattle and especially the copro-antigen ELISA is far from complete. The faecal egg count test, is commonly used in practice but can only detect patent infections. The serum antibody ELISA has the limitation of providing information on exposure rather than current infection but can detect exposure even at pre-patent stages of infection [11]. On the other hand the copro-antigen ELISA, which detects *F. hepatica* excretory-secretory antigens in faeces, is reported to detect early stages of infection without the limitation of giving positive results due to past exposure [12, 13]. This test has been evaluated by different research groups with varying results in sheep, but little has been reported on its performance in cattle [14].

Furthermore, inspection of livers of cattle slaughtered in abattoirs across Europe for signs of liver fluke is mandatory according to Regulation (EC) No 854/2004. In a previous study in Switzerland, Rapsch et al. (2006) [15] estimated the sensitivity of abattoir liver inspection to be 63.2%. Such estimates are expected to vary between countries, hence it is important to be able to obtain estimates specific to each country. Lastly, detailed liver necropsy techniques including gall bladder egg counts are available for research purposes, but impractical and expensive for routine use. These are expected to be extremely sensitive, even though there is still a window of error in case of very early stage infections. Moreover they can provide information on the severity of infection according to the degree of damage, as well as the fluke burden.

In this study we have used the above diagnostic tests on samples taken from Scotbeef, one of Scotland’s largest red meat abattoirs, receiving animals from all around Scotland, northern England and Northern Ireland in an attempt to improve our knowledge on the performance of these diagnostic tests in the UK setting. More precisely this analysis aims to estimate: i) the performance of meat inspection as a tool for diagnosis of *F. hepatica* infection; and ii) the performance of liver necropsy, serum antibody ELISA, the copro-antigen ELISA and faecal egg count diagnostic tests.

## Materials and Methods

### Abattoir Based Sampling

Samples were collected from Scotbeef Limited, Scotland’s largest red meat abattoir during three sampling periods. Sampling period A (June-July 2013) will be referred to as “summer 2013”. Sampling period B (January-beginning of March 2014) will be referred to as “winter 2014”. Lastly, sampling period C, which took place between the end of August 2014 and October 2014 will be referred to as “autumn 2014”. Each period consisted of six sampling days, one per week and 32-36 animals were sampled each time. The day and number of animals sampled each day were constrained by logistics. We used systematic sampling, collecting samples from one cattle in every 10 slaughtered to allow time for processing and represent animals slaughtered during the whole day. Animals to be sampled were clearly labeled at the time of bleeding and labels were maintained at all sampling stages to ensure that the correct samples were taken. Samples included blood, faecal samples as well as whole livers and gall bladders from each animal. Whole livers and gall bladders were stored at 4°C and were analysed within 72 and 96 hours respectively. Blood samples were stored at 4°C for 24 hours before sera were obtained and stored at -20°C. 2g of faeces were stored at -20°C, while the rest was stored at 4°C for egg counting which took place within a week post sampling.

### Diagnostics Tests

#### Necropsy

**a) Liver dissection** Livers were laid out on a tray and incisions parallel to and approximately 1 cm apart from the meat inspector’s incisions were made. Grades from 0 to 3 (no, mild, moderate, severe) were given in terms of signs of fibrosis; 0—no signs of fibrosis, 1—mild focal fibrosis, 2—severe local fibrosis or mild generalised fibrosis, 3—severe local fibrosis with calcified biled ducts or severe generalised fibrosis. Fibrosis scores were assigned before slicing the liver further in order to mimic what a meat inspector would be able to see on the offal line in the abattoir. The liver was cut into 1-2 cm slices thick and each slice was squeezed in order to collect flukes present. The slices were then placed in a bucket containing hot water for approximately 30 minutes. Water contents were then poured through 200*μ*m sieves and inspected to retrieve flukes. Each slice was squeezed so that fluke exited the bile ducts, rinsed with water flowing in the bucket and discarded. Water remaining in the bucket was poured through 200*μ*m sieves and inspected to retrieve remaining flukes. Flukes were then counted and stored in formalin. The total number of flukes was based on the number of whole flukes plus the number of anterior or posterior fluke parts depending on which one was greater [16, 17].

**b) Gall bladder egg count** Gall bladder contents were sieved through a series of 250 and 150*μ*m sieves and collected in a measuring flask. The content was allowed to sediment for 3 minutes, excess liquid was removed and the remaining liquid was agitated and poured into a narrow bottomed glass. Water was added to the flask and poured into the glass to ensure no eggs remained in the flask. This process was repeated and liquid was poured in a 15ml falcon tube and allowed to sediment for 3 minutes. The sediment was collected in a petri dish, one drop of 0.5% methylene blue was added and all the eggs on the plate were counted using a stereoscopic dissecting microscope [13].

An animal was classified as positive for liver necropsy when 1 or more parasites were found in the liver and/or 1 or more eggs were found in the gall bladder.

**Faecal egg count (FEC)** The faecal sample was mixed using a spatula and 5g were weighed out in a measuring cylinder. Water was added up to the 40ml mark and contents were mixed using a stirring rod. Contents were sieved through a coffee strainer and collected in a 250ml beaker for removal of coarse faecal material. The contents were then sieved through a 150*μ*m sieve, collected into a narrow bottomed glass and allowed to sediment for 3 minutes. Excess liquid was syringed off and sediment was transferred into a 15ml falcon tube and allowed to sediment for 3 minutes. Excess liquid was syringed off and the sediment was transferred onto a petri dish. One drop of 0.5% methylene blue was added and all the eggs on the plate were counted using a stereoscopic dissecting microscope [18]. A sample was classified as positive when 1 or more eggs were found in the sample.

**Copro antigen ELISA (cELISA)** Faecal samples were tested for the presence of excretory-secretory antigens using the commercially available *Fasciola hepatica* antigen ELISA kit (Bio-X Diagnostics, Belgium). The test was performed following the manufacturer’s instructions [12] and results were expressed as the sample optical density (OD) as a percentage of the mean positive control OD.

$$\begin{array}{c} \text{Percent}\;\text{positive}=\frac{\text{Sample}\;\text{OD}}{\text{Mean}\;\text{positive}\;\text{control}\;\text{OD}}*100 \end{array}$$

Samples were classified as positive or negative according to the cut-offs provided by the manufacturer for each batch.

**Serum antibody ELISA (sELISA)** Serum samples were analysed using the excretory/secretory (ES) antibody ELISA developed by the Liverpool School of Tropical Medicine [11]. The procedure described by Salimi-Bejestani et al (2005) [11] was performed with the following modifications:

- 1:8000 monoclonal mouse anti-bovine IgG conjugate (AbD Serotec, Bio-Rad Laboratories Inc, Hertfordshire, UK) was used.
- A new positive control was used so the equation used for calculating the results was slightly varied to obtain comparable results to previous controls. The percent positive (PP) value was obtained by the quotient of the mean sample OD (based on two duplicates) divided by the mean positive control OD (four duplicates), which was then multiplied by 111 instead of 100 to account for the new positive control as suggested by the test developers at Liverpool (Prof. D. Williams, 2014, pers.comm., 1 Dec).
  $$\begin{array}{c} \text{Percent}\;\text{positive}=\frac{\text{Mean}\;\text{test}\;\text{sample}\;\text{OD}}{\text{Mean}\;\text{positive}\;\text{control}\;\text{OD}}*111 \end{array}$$

Samples were classified as positive if they had a PP greater or equal to 10.

**Liver inspection by the Meat Hygiene Service (MHS)** The final test included in this analysis is liver inspection carried out at the abattoir by the Meat Hygiene Service. According to the manual for official controls, liver inspection requirements include visual inspection, palpation and incision of the gastric surface of the liver [19]. Livers with signs of liver fluke related pathology then have to be condemned. At Scotbeef, since 2012, MHS decision regarding liver condemnation is recorded as ‘Active’, ‘Historic’ or ‘No fluke’. ‘Active’ is roughly defined as livers in which parasites were seen, while ‘Historic’ describes livers with liver fluke related pathology but no signs of current infection. Both ‘Active’ and ‘Historic’ livers have to be condemned. This is unlike most UK abattoirs and for the purposes of this analysis we will be using the standardised classification, considering ‘Active’ and ‘Historic’ livers as positive and ‘No fluke’ livers as negative.

**Forestomach inspection for presence of rumen fluke** During the second and third sampling seasons of the study (autumn and winter 2014) forestomachs of sampled animals were inspected for the presence of rumen fluke parasites. This procedure was carried out as part of a separate study [20], but results will be used here to assess the copro-antigen and the serum-antibody ELISAs for cross-reactivity with rumen fluke.

### Statistical Analysis

**A. The No Gold Standard (NGS) estimation of diagnostic test performance** NGS, introduced by Hui & Walter [21], is a latent class approach to the evaluation of diagnostic tests when a “gold standard” is not available. The Bayesian version incorporates prior knowledge by specifying prior distributions for test properties and prevalence. If no prior information is available, vague, uniform priors are set. Probabilities of all the possible combinations of test outcomes conditional on the unknown disease status are specified using the sensitivity (Se) and specificity (Sp) of each test and the prevalence (p) of each sub-population, in this case periods “summer 2013”, “winter 2014” and “autumn 2014” [15, 22]. Animals can be positive or negative for each of the five tests included in this analysis so there are 2^5^ (i.e. 32) possible combinations of test results. Hence, for each sub-population the counts of animals (O_i_) of each combination of test results, in this case 32 (S) combinations for the five tests (T), follow a multinomial distribution [23, 24]:

$$\begin{array}{c} {\text{O}}_{i}|{\text{Se}}_{j},{\text{Sp}}_{j},{\text{p}}_{i}∼\text{Multinomial}\left({\text{Pr}}_{i},{\text{n}}_{i}\right)\;\;\text{for}\;\;\text{i}=1,2,\ldots ,\text{S}\;\;\text{and}\;\;\text{j}=1,2,\ldots ,\text{T} \end{array}$$

where Pr_i_ is the probability of observing the *i*th combination of test results.

**Examples of how to specify two such probabilities are shown below:**

1. Probability of obtaining a positive result in all five tests
  Pr(*T*_1_+, *T*_2_+, *T*_3_+, *T*_4_+, *T*_5_+) = Se_1_Se_2_Se_3_Se_4_Se_5_p_*i*_ + (1 − Sp_1_)(1 − Sp_2_)(1 − Sp_3_)(1 − Sp_4_)(1 − Sp_5_)(1 − p_*i*_)
2. Probability of obtaining a positive result in the first four tests and a negative result in the fifth test
  Pr(*T*_1_+, *T*_2_+, *T*_3_+, *T*_4_+, *T*_5_−) = Se_1_Se_2_Se_3_Se_4_(1 − Se_5_)p_*i*_ + (1 − Sp_1_)(1 − Sp_2_)(1 − Sp_3_)(1 − Sp_4_)Sp_5_(1 − p_*i*_)

The ratio of acute versus chronic infection is expected to be different, according to the known lifecycle of the parasite, between the three different times of the year which may affect the sensitivities and/or specificities of certain tests. Therefore, different estimates for the sensitivities of FEC, the copro-antigen and the serum antibody ELISA tests were obtained for each season as well as the specificity of the serum antibody ELISA. This was done for two reasons. Firstly, as shown by Toft et al (2005) [23], if estimates vary between sub-populations the combined estimate will be biased towards the estimate supported by most data i.e the one from the sub-population with the highest prevalence. Secondly, this can provide information on which tests are more appropriate at different times of the year.

#### Model Assumptions

1. Tests are conditionally independent. In other words, the misclassification errors of each test are unrelated conditional on the true disease status of the animal. For example, the probability of a truly diseased animal testing positive in test 2 (sensitivity), is not altered by the result of test 1 [25, 26]. There are various models for accounting for conditional dependence. In this case we have used the model suggested by Vacek (1985) as described below [23, 25]. Ten models including covariance terms (*γ*_*Se*_ and *γ*_*Sp*_) for one combination of two tests at a time were specified in order to inspect the effect of adjusting for covariance for each test combination on the sensitivity and specificity estimates of all tests. For example:
  - Probability of obtaining a positive result in all five tests accounting for covariance between tests 1 and 2
    Pr(*T*_1_+, *T*_2_+, *T*_3_+, *T*_4_+, *T*_5_+) = (Se_1_Se_2_+*γ*_Se_)Se_3_Se_4_Se_5_p_*i*_ + ((1 − Sp_1_)(1 − Sp_2_)+*γ*_Sp_)(1 − Sp_3_)(1 − Sp_4_)(1 − Sp_5_)(1 − p_*i*_)
  - Probability of obtaining a negative result in the first test and a positive result in all other tests accounting for covariance between tests 1 and 2
    Pr(*T*_1_−, *T*_2_+, *T*_3_+, *T*_4_+, *T*_5_+) = ((1 − Se_1_)Se_2_ − *γ*_Se_)Se_3_Se_4_Se_5_p_*i*_ + (*Sp*_1_(1 − *Sp*_2_) − *γ*_Sp_)(1 − Sp_3_)(1 − Sp_4_)(1 − Sp_5_)(1 − p_*i*_)
  - Probability of obtaining a negative result in the first two tests and a positive result in all other tests accounting for covariance between tests 1 and 2
    Pr(*T*_1_+, *T*_2_+, *T*_3_+, *T*_4_+, *T*_5_+) = ((1 − Se_1_)(1 − Se_2_) + *γ*_Se_)Se_3_Se_4_Se_5_p_*i*_ + (Sp_1_Sp_2_+*γ*_Sp_)(1 − Sp_3_)(1 − Sp_4_)(1 − Sp_5_)(1 − p_*i*_)
2. Test sensitivities and specificities are constant between populations.
3. Prevalences vary between populations
  The original Hui & Walter model contained two tests and two populations. Assumptions 3 and 4 were there to ensure that there are enough degrees of freedom to ensure the model’s identifiability. As liver fluke infection levels vary throughout the year and between years, we were able to assume that the prevalence will vary between the three sampling seasons. Additionally, according to Toft et al (2005) when three or more tests are compared one population is enough [23] to have sufficient degrees of freedom. As this model is an adaptation of the original model, with three sub populations and five tests, we ensure that we have enough degrees of freedom to be able to allow the stated sensitivities and specificities to vary between sub-populations and to include covariance terms for one combination of tests at a time.

**MCMC diagnostics** Markov chain Monte Carlo (MCMC) chain convergence was assessed by visual inspection of the three sample chains using trace and Gelman-Rubin diagnostic plots for each variable in the model [27]. A correlation matrix of each chain was plotted to check for high correlation between variables.

**Priors** As a Bayesian framework is used in this analysis, prior distributions were specified for the prevalence of each sub-population, sensitivities and specificities of each test. Vague, uniform priors with an interval between 0 and 1 were used for the prevalence of each sub-population.

$$\begin{array}{c} \text{p}∼dbeta(1,1) \end{array}$$

Similarly for the sensitivities and most of the specificities of evaluated tests, a wide distribution with an interval between 0 and 1 was used to reflect the fact that there is scarce knowledge on the performance of most of these tests in a real life scenario.

$$\begin{array}{ccc} \text{Se} & ∼ & dbeta(2,1)\\ \text{Sp} & ∼ & dbeta(2,1) \end{array}$$

Liver necropsy was the only test where the prior distribution given for the specificity was highly informative. As mentioned before an animal was classified as positive for liver necropsy when either at least one fluke was found in the liver and/or when at least one egg was seen in the bile sample. It is therefore very unlikely that an animal can be wrongly classified as positive as liver flukes are easily identifiable and no other eggs similar to *Fasiola hepatica* eggs are expected to be seen in the bile. In a previous study by Rapsch et al (2006) [15] a similar test was assigned a specificity of 1 for the reasons explained. In order to account for the possibility of egg sequestration in the gall bladder for up to three weeks post treatment [28] we chose the following prior distribution instead.

$$\begin{array}{c} {\text{Sp}}_{\text{liver}\;\text{necropsy}}∼dbeta\left(9,1\right) \end{array}$$

The analysis was repeated using priors dbeta(1,1) for the Se and Sp of all tests to assess the effect of priors.

Priors for the covariance variables, *γ*_Se_ and *γ*_Sp_, were uniform distributions using the following maximum and minimum limits [22, 29].

$$\begin{array}{ccc} \left({\text{Se}}_{1}-1\right)\left(1-{\text{Se}}_{2}\right) & \leq & \gamma_{\text{Se}}\leq min\left({\text{Se}}_{1},{\text{Se}}_{2}\right)-{\text{Se}}_{1}{\text{Se}}_{2}\\ \left({\text{Sp}}_{1}-1\right)\left(1-{\text{Sp}}_{2}\right) & \leq & \gamma_{\text{Sp}}\leq min\left({\text{Sp}}_{1},{\text{Sp}}_{2}\right)-{\text{Sp}}_{1}{\text{Sp}}_{2} \end{array}$$

**Model implementation** The model was implemented in JAGS [30], a software which uses MCMC simulations to construct posterior distributions for the analysis of Bayesian hierarchical models. JAGS was run within R (Version 3.0.3) [31] using the *rjags* package [32]. The first 20,000 iterations were discarded as burn-in and the following 20,000 iterations were used to construct the posterior distributions. The model specification is included in “S1 R script”. R Package *coda* [27] was used to carry out MCMC diagnostics and package *corrplot* [33] was used to visualize the correlation matrix between variables. The results were plotted using *ggplot2* [34]. A map showing the distribution of sampled animals was plotted using *ggmap* [35] and the map tiles were sourced from Stamen Design (using data by OpenStreetMap), which are freely available under CC BY 3.0 license.

**Positive and Negative Predictive Values** Sensitivity and specificity estimates report diagnostic test validity however positive (PPV) and negative predictive values (NPV) are the appropriate measure for interpreting tests in a specific population. They are the probability that a test positive or negative animal is truly positive or negative respectively. This is more easily interpreted by both farmers and vets, but its value depends on the true prevalence of the disease in the population [36]. Based on the Bayes formula [37], presented below, one can estimate the predictive values using estimates for sensitivity (Se), specificitiy (Sp) and the true population prevalence (p) [38].

$$\begin{array}{ccc} \text{PPV} & = & \frac{\text{Se}*p}{\left(\text{Se}*\text{p})+(1-\text{Sp})*(1-\text{p}\right)}\\ \text{NPV} & = & \frac{\text{Sp}*(1-p)}{(\text{Sp}*(1-\text{p}))+(1-\text{Se})*\text{p}} \end{array}$$

PPVs and NPVs of the MHS liver inspection and FECs were calculated using the Se and Sp estimated by the NGS model over a range of possible prevalences to demonstrate this.

## Results

### Descriptive statistics

**Fig 1:**
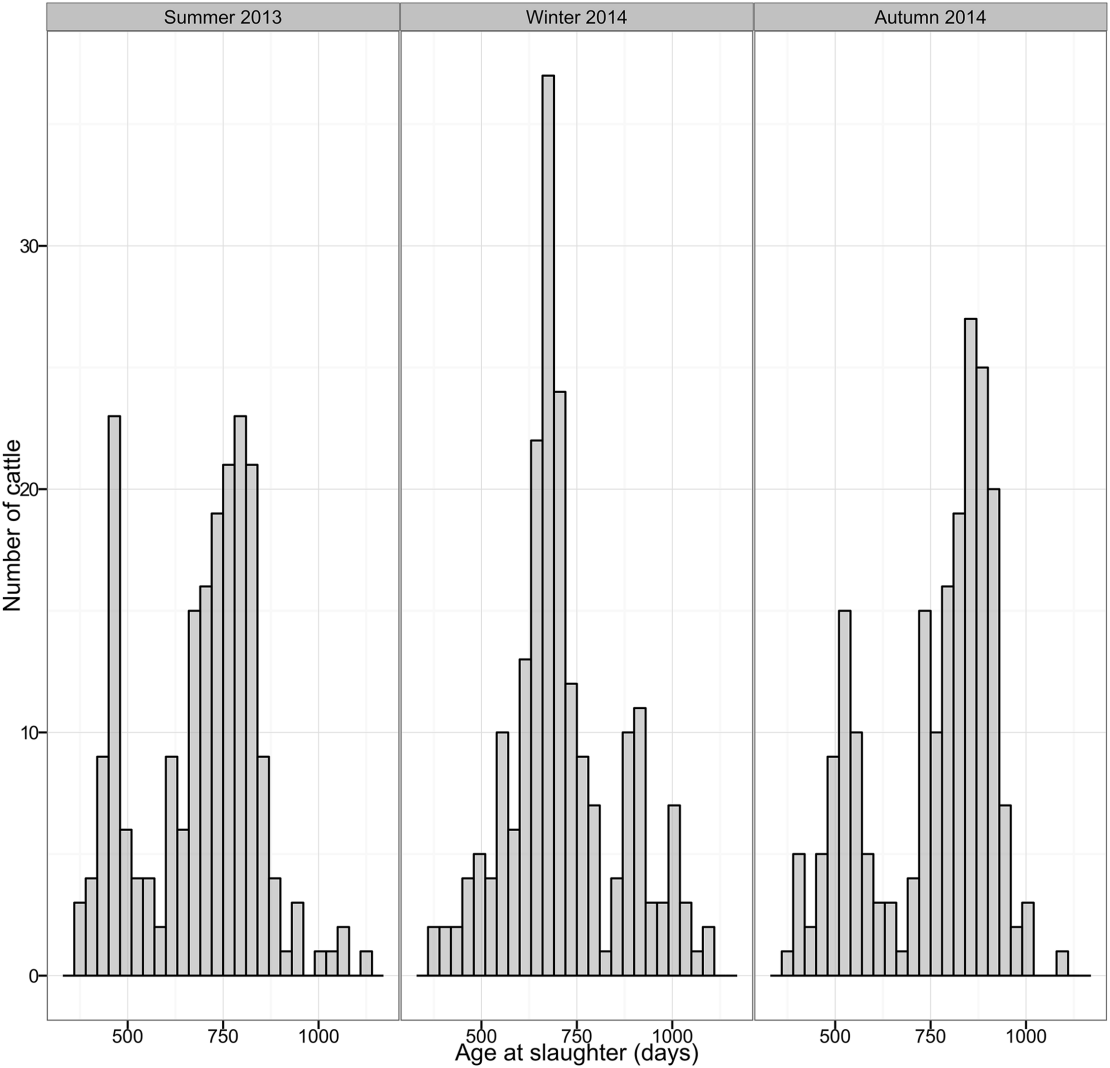
Distribution of cattle age per period. The age of cattle sampled ranged from 369 to 1121, with a mean of 720 days old.

**Fig 2:**
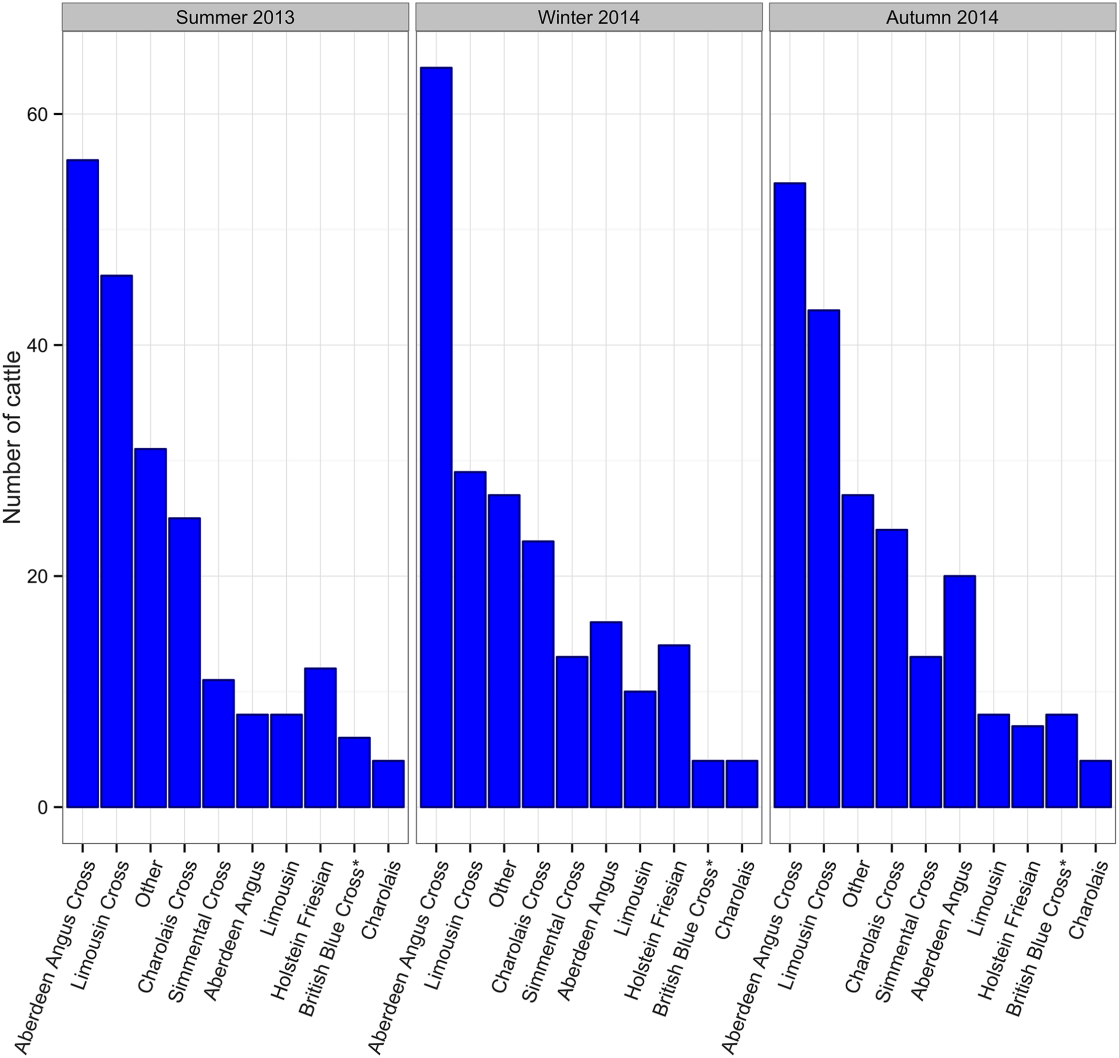
Distribution of cattle breed per period. Cattle sampled were of a range of different breeds found in the UK. 175 cattle were Aberdeen Angus cross, 118 were Limousin cross, 73 were Charolais cross, 48 were Aberdeen Angus, 37 were Simmental, 33 Holstein Friesian, 26 Limousin, 18 British Blue cross, 12 Charolais and 85 were of other less common breeds.

**Fig 3:**
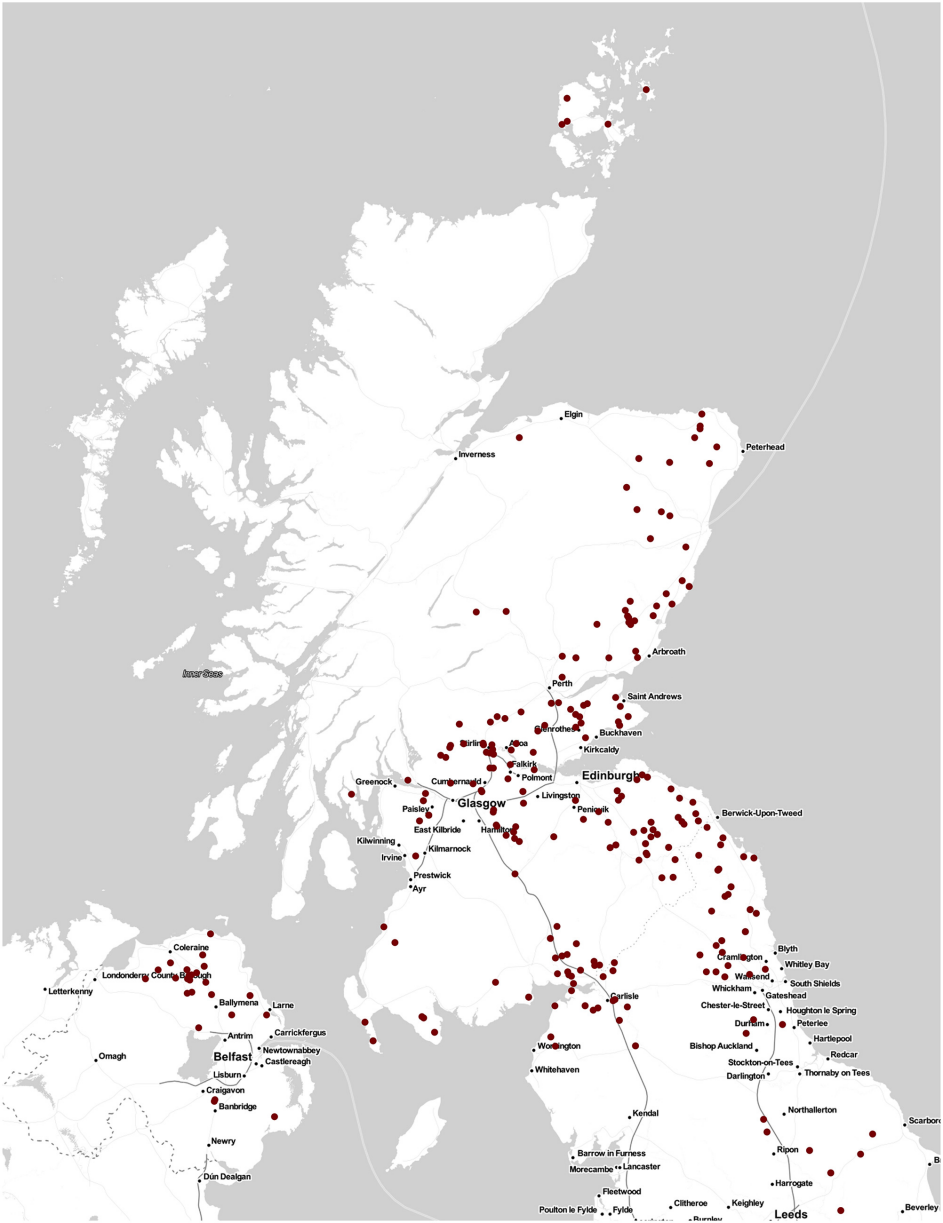
Geographical distribution of cattle sampled. Samples used in this study were taken from Scotbeef, one of Scotland’s largest red meat abattoirs, receiving animals from all around Scotland, northern England and Northern Ireland. Figure shows the distribution of cattle sampled i.e the geographical distribution of the general population of cattle slaughtered at the abattoir was well represented. The map was plotted using R package *ggmap* [35] using tiles sourced from Stamen Design (using data by OpenStreetMap).

In total, 619 cattle were sampled, 207 during summer 2013, 204 during winter 2014 and 208 during autumn 2014. Cattle age ranged from 369 to 1121 days old (Fig 1) and cattle of a variety of breeds were sampled as shown in Fig 2. As Fig 3 shows, cattle sampled came from Scotland, northern England and Northern Ireland i.e the geographical distribution of the general population of cattle slaughtered at the abattoir was well represented. Samples from every cattle were tested with the five tests mentioned.

### Diagnostic test results

**Fig 4:**
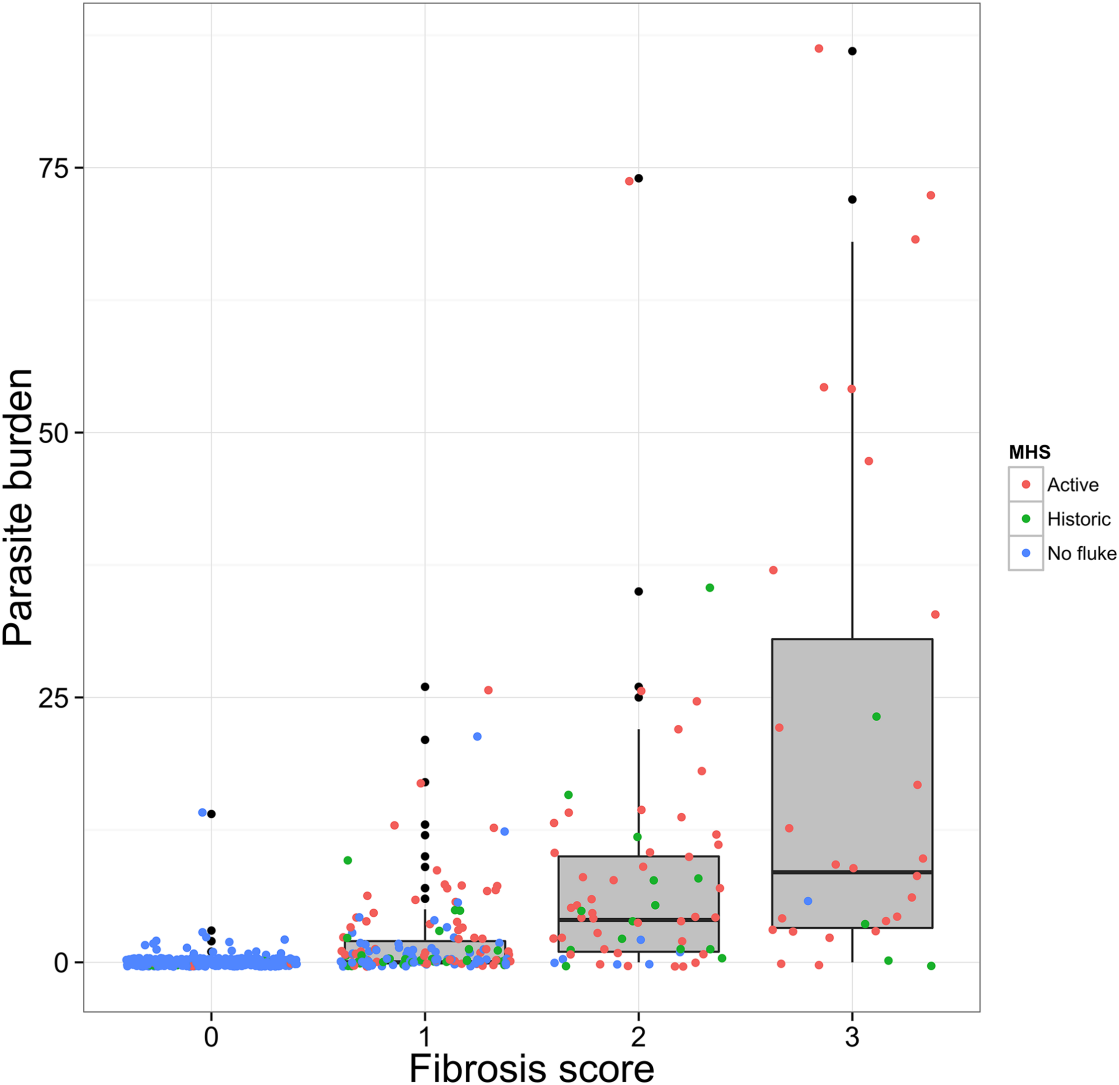
Distribution of parasite counts by fibrosis score and MHS classification. Figure shows the distribution of parasite burden per fibrosis score as recorded during liver necropsy. Among livers where flukes were found, parasite burden ranged from 1 to 86 parasites, with a mean of 8.5 and a median of 4. A fibrosis score was assigned based on a presentation of the liver mimicking the one presented to the MHS. The colour of the points shows the decision taken by the MHS during liver inspection at the abattoir.

Table 1 shows the binary results of each test per sampling period. Fig 4 shows the distribution of parasite burden per fibrosis score as recorded during liver necropsy. Among livers where flukes were found, parasite burden ranged from 1 to 86 parasites, with a mean of 8.5 and a median of 4. As previously described a fibrosis score was assigned based on a presentation of the liver mimicking the one presented to the MHS. The colour of the points shows the decision taken by the MHS during liver inspection at the abattoir. Higher fibrosis scores appear to have higher parasite burden, but it is also important to note that livers with no signs of fibrosis, that were also not rejected at the abattoir were found to have parasites. Furthermore, many livers which were classified as “Historic” by the MHS (green) were found to have parasites. Lastly, livers classified as “Active” by the MHS (red) appear to be spread evenly among fibrosis scores 1 to 3, while there were a few livers with a fibrosis score 0 which were classified as “Active”. This might mean that what was presented at the liver necropsy was not always the same as what was seen by the MHS.

**Table 1:** Proportions of test positives for each test and number of animals sampled.

	summer 2013	winter 2014	autumn 2014	Overall
**Number sampled**	207	204	208	619
**MHS inspection**	0.32	0.29	0.25	0.29
**Necropsy**	0.39	0.33	0.23	0.32
**cELISA**	0.29	0.25	0.18	0.24
**FEC**	0.31	0.25	0.13	0.23
**sELISA**	0.35	0.36	0.37	0.36

### Estimates of diagnostic test sensitivity and specificity

**Fig 5:**
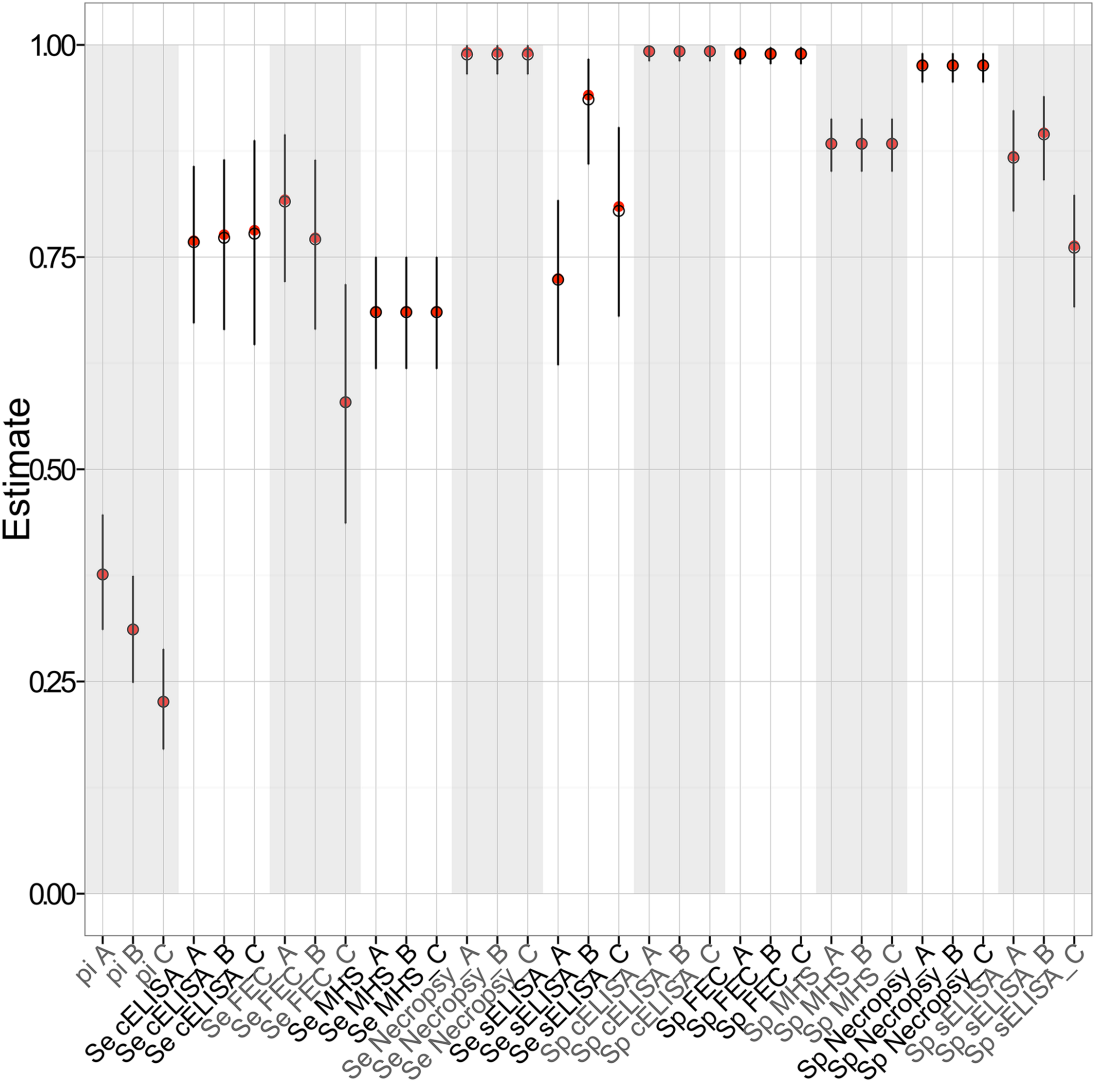
Mean posterior estimates and 95% BCIs. Estimates of the prevalence (pi), sensitivity (Se) and specificity (Sp) for each period (summer 2013 (A), winter 2014 (B), autumn 2014 (C)).

Fig 5 is a plot of mean estimates and 95% Bayesian Credible Interval (BCI) for each model parameter. The precise mean estimates and 95% BCIs for each variable are shown in Table 2. *F. hepatica* infection prevalence during summer 2013, winter 2014 and autumn 2014 sampling periods was estimated to be 0.38, 0.31 and 0.23 respectively. Liver necropsy was, as expected, a near perfect test with a sensitivity estimate of 0.99 and a specificity of 0.98. Liver inspection by the abattoir Meat Hygiene Service had a sensitivity estimate of 0.68 and a specificity of 0.88. The sensitivity estimates of the copro-antigen ELISA were allowed to vary between seasons, but were estimated as 0.77 for all three sampling seasons. cELISA was estimated to have a very high specificity of 0.99. The Faecal Egg Count sensitivity values varied greatly between sampling seasons and were estimated as 0.81, 0.77 and 0.58 respectively. The test was shown to be highly specific, 0.99. Lastly, both the sensitivity and the specificity of the serum antibody ELISA were allowed to vary between seasons. Sensitivity estimates varied between seasons with the mean sensitivity estimate being much higher during the winter sampling, 0.94, compared to 0.72 and 0.80 during the summer and autumn sampling periods respectively. Similarly the mean specificity estimate during the autumn sampling of 0.76 was comparatively lower than summer and winter estimates which were 0.87 and 0.89 respectively. The exact data used for this model can be found in “S1 Table”.

**Table 2:** Mean estimates and 95% BCIs of the prevalence and test sensitivity and specificity per period.

Estimate (Season)	Mean	2.5% BCI	97.5% BCI	Estimate (Season)	Mean	2.5% BCI	97.5% BCI
**Prevalences**							
Summer 2013 (A)	0.38	0.31	0.45				
Winter 2014 (B)	0.31	0.25	0.38				
Autumn 2014 (C)	0.23	0.17	0.29				
**Sensitivities**				**Specificities**			
MHS inspection	0.68	0.61	0.75	MHS inspection	0.88	0.85	0.91
Necropsy	0.99	0.96	1	Necropsy	0.98	0.96	0.99
cELISA (A)	0.77	0.67	0.86	cELISA	0.99	0.98	1
cELISA (B)	0.77	0.67	0.87				
cELISA (C)	0.77	0.64	0.88				
FEC (A)	0.81	0.72	0.9	FEC	0.99	0.98	1
FEC (B)	0.77	0.66	0.86				
FEC (C)	0.58	0.43	0.72				
sELISA (A)	0.72	0.62	0.82	sELISA (A)	0.87	0.8	0.92
sELISA (B)	0.94	0.86	0.98	sELISA (B)	0.89	0.84	0.94
sELISA (C)	0.8	0.69	0.91	sELISA (C)	0.76	0.69	0.82

### Model checking

Supporting information contain figures to demonstrate the results of checking for conditional dependence, the effect of priors and correlation between model variables, respectively. As shown in “S1 Fig”, there are no major differences in estimates when accounting for covariance for the different combinations of tests and the model with no covariance terms. It was therefore justifiable to use a final model with no covariance terms. Furthermore, “S2 Fig”, show a comparison of prior and posterior distribution which reveals that results are mainly informed by the data. This is further supported by “S3 Fig”, which presents a comparison between the results presented in the paper and the results of the same model run using non-informative priors for the sensitivities and specificities of all tests, where results do not appear to be altered. Lastly, “S4 Fig” presents the cross correlation plots between the parameters included in the model showing that there is no obvious strong correlation between any combination of parameters.

### Predictive Values of diagnostic tests

**Fig 6:**
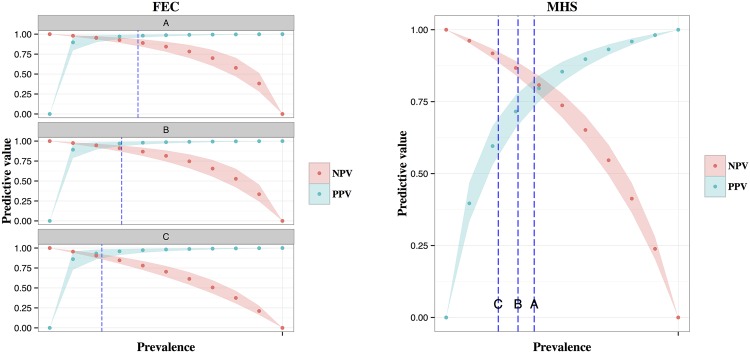
Predictive values of a) FEC and b) MHS over a range of prevalences. Prevalence estimates for each sampling period are shown by dotted lines.

Fig 6 show the positive and negative predictive values of the MHS liver inspection and Feacal egg counts respectively, over a range of prevalences. Estimates for FEC sensitivity was allowed to vary over the 3 sampling seasons hence 3 plots are presented. Prevalence estimates of the 3 sampling periods are shown by dotted lines. It is important to note how predictive values change according to the population prevalence. Additionally, when the PPV values of the two tests are compared at low prevalence levels it is clear that PPV of FEC is higher and varies less than the PPV of MHS due to a much higher specificity estimate for FEC.

## Discussion

The main aims of this study was the evaluation of the performance of tests available for the diagnosis of *F. hepatica*. The no gold standard approach introduced by Hui & Walter [21] was used within a Bayesian framework in order to compare the binary results of the five diagnostic tests.

Estimates of sensitivity and specificity for liver necropsy were 0.99 (95% BCI 0.96-1.00) and 0.98 (95% BCI 0.96-0.99) respectively. Liver necropsy is not readily used for disease diagnosis by veterinarians as it is a very time consuming procedure and it can only be carried out post mortem. Its role in this study was to provide a measure of infection and fibrosis levels to better describe the sample. Additionally, as a test previously used as a gold standard in assessments of *F. hepatica* diagnostic tests [39] it was expected to provide near perfect results and therefore be highly informative. A gold standard analysis was not chosen due to the possibility of gall bladder egg sequestration in animals where infections has been successfully treated causing false positive results and very early infections being difficult to detect due to the small size of flukes causing false negative results. The results of this study show that liver necropsy has a near perfect sensitivity and a very high specificity and must have contributed greatly in the evaluation of the rest of the tests by our model.

Liver inspection is routinely carried out at the abattoir according to Regulation (EC) No 854/2004. The only previously reported estimate of its sensitivity in a European setting identified by the author was from a study in Switzerland by Rapsch et al. (2006) [15] which was 63.2%. In the current study the sensitivity estimate of liver inspection appeared to be lower than all other diagnostic tests, except that of FEC during Autumn 2014. Similarly, specificity appeared to be similar to the serum antibody ELISA, but lower than all other tests. More precisely the sensitivity was estimated to be 0.68 (95% BCI 0.61-0.75) and the specificity 0.88 (95% BCI 0.85-0.91). Estimates for meat inspection are expected to vary between countries and potentially between abattoirs. It is therefore relevant to report estimates for liver inspection from one of the biggest abattoirs in Scotland as this can provide a way to more accurately estimate the prevalence of *F. hepatica* infection in the UK accounting for imperfectness of this technique. Additionally, liver inspection can provide a useful and practical tool for evaluation of the effectiveness of health planning programmes used on farms. In this setting it is possibly more intuitive to use positive and negative predictive values, which can readily be estimated based on population prevalence as shown in the results section.

Mezo et al. (2004) presented a new copro-antigen ELISA which was reported to have a sensitivity of 100% in detecting cattle with fluke burden of two or more parasites and be highly specific with no cross reactivity with parasites including *Moniezia*, *Dicrocoelium*, *Echinococcus* and *Paramphistomum cervi* [12, 14]. This ELISA is commercially available by Bio-X Diagnostics in Belgium. The protocol used in the commercial test is a considerable modification of the original, and its performance in the field setting has been poorly assessed, especially in cattle.

In this study sensitivity estimates of the copro-antigen ELISA were allowed to vary between seasons, but were in fact very similar. They were estimated to be 0.77 (95% BCI 0.67-0.86), 0.77 (95% BCI 0.67-0.87), 0.77 (95% BCI 0.64-0.88) during summer 2013, winter 2014 and autumn 2014 sampling periods respectively. These estimates were considerably lower compared to Charlier et al. (2008) [39] who reported a sensitivity of 94%. This might be because liver necropsy without detection of eggs in the gall bladder was used as the gold standard, potentially missing a proportion of infected animals and therefore overestimating the sensitivity. Additionally, a lower cut-off than the one recommended in the protocol was used which might increase the sensitivity. Our estimate was similar to that of Palmer et al (2014) [40] who estimated the sensitivity to be 0.80 when the cut-off recommended by the manufacturer was used. When using a lower cut-off Palmer et al (2014) estimated the sensitivity to be 87%.

The specificitiy of copro-antigen ELISA was estimated to be 0.99 (95% BCI 0.98-1.00). This is comparable to Palmer et al (2014) who estimated the specificity to be 1 using the manufacturer’s cut off and >99% using their own cut off [40]. On the contrary, Charlier et al (2008) estimated the specificity to be 93%. This might be a result of their cut-off adaptation. As the cut off adjustment used by Palmer et al. (manufacturer’s cut off multiplied by 0.67) provided greater improvement in the test performance, the model was rerun using the modified cut-off for the cELISA. Sensitivity was estimated as 0.80 (95% BCI 0.71-0.89), 0.85 (95% BCI 0.75-0.93), 0.87 (95% BCI 0.76-0.95) during summer 2013, winter 2014 and autumn 2014 sampling periods respectively. The specificity remained 0.99 (95% BCI 0.98-1.00) confirming that this cut-off modification can improve test sensitivity without compromising specificity. Estimates regarding the other four tests were not altered (results not shown).

Gordon et al. (2013) [41] identified rumen fluke from a range of cattle and sheep samples across the UK to be *Calicophoron daubneyi* instead of *P. cervi* which was previously thought to be the species found in the UK. Even though lack of cross-reactivity with *P. cervi* has already been reported [42], this emphasises that it is important to also check for cross-reactivity of cELISA with *C. daubneyi*. In our study, during the second and third sampling seasons 53 cattle with negative liver necropsy results were found to have at least one fluke in the rumen. None of those samples had a positive copro-antigen result (using both manufacturer’s and adjusted cut-off). Rumen flukes collected have not been speciated, but based on the findings of Gordon et al. (2013) it is reasonable to assume that a great proportion of those were *C. daubneyi*. This suggests that cELISA does not cross react with this parasite in cattle, which agrees with the results of a similar comparison with cELISA in sheep [41]. This is becoming increasingly important in the UK as levels of rumen fluke infection appear to be rising and will further complicate fasciolosis control.

Diagnosis of *F. hepatica* infection by detection of eggs in faecal samples has been around for decades and various protocols exist. The main drawbacks, of this otherwise easy to learn method, are that by definition it can only diagnose patent infections and that it is time consuming and therefore costly or undercharged. It is generally accepted that the specificity of faecal egg counting is almost perfect. In the UK this might be compromised by the increasing levels of rumen fluke infection as the eggs are of similar shape [41], even though the trained eye should be able to discriminate between the two kinds of eggs as they are of different colour. As vets and technicians become more aware of the increasing chance of finding rumen fluke eggs in faeces this problem is expected to be reduced. On the other hand, the sensitivity of the test has been reported to vary from well below 50% to moderate values and depends on various factors mainly based on the protocol used, for example volume of faeces [15, 39] and levels of infection in the population [43]. In the current context FEC sensitivity was estimated to be 0.81 (95% BCI 0.72-0.90), 0.77 (95% BCI 0.66-0.86) and 0.58 (95% BCI 0.43-0.72) during summer 2013, winter 2014 and autumn 2014 respectively. As expected the specificity was close to perfect and comparable to the copro-antigen ELISA (0.99, 95%, BCI 0.98-1.00). The sensitivity of FEC was shown to be comparable to cELISA during the first two sampling seasons, while it dropped significantly during autumn 2014. This shows that FEC still remains a very useful test during periods where infection is expected to be mainly chronic, and even superior to antibody ELISA tests as it has a higher specificity. As shown here it is important to remember that when recent infections are expected, for instance at the start of a new liver fluke season, this test performs a lot worse than other tests due to its inability to detect pre-patent infections.

The last test evaluated in this study was the excretory/secretory antibody ELISA developed by the Liverpool School of Tropical Medicine [11]. This is the only test included that is developed to also detect past exposure to the parasite. Therefore, both the sensitivity and the specificity were allowed to vary between seasons. Sensitivity appeared to be much higher during the winter sampling, 0.94 (95% BCI 0.86-0.98) when compared to 0.72 (95% BCI 0.62-0.82) and 0.80 (95% BCI 0.69-0.91) during the summer and autumn sampling periods respectively. It was particularly interesting to see whether the false positive rates differed as well. Indeed, specificity during the autumn sampling was estimated to be 0.76 (95% BCI 0.69-0.82), which was comparatively lower than summer and winter estimates of 0.87 (95% BCI 0.80-0.92) and 0.89 (95% BCI 0.84-0.94) respectively. Serum antibody ELISA tests for the diagnosis of *F. hepatica* have been around for decades and have various reported sensitivities and specificities ranging from 91.7% to 100% and 94.6% to 100% respectively [44]. The ELISA used in this study is not commercially available and was first presented by Salimi-Bejestani et al. in 2005 with a sensitivity of 98% and a specificity of 96%. For their test evaluation they used FEC positive cattle, while their negative samples came from zero-grazed cattle of no known previous exposure to the parasite. Our sensitivity estimates are much lower and this is thought to be because the test was evaluated using an abattoir random sample of a range of levels of infection, including ones not detectable by FEC. Similarly, our specificity estimates are lower than previously reported. This is believed to be a result of the inability of antibody ELISAs to distinguish between current and previous exposure as it is highly possible that our sample included animals that were previously infected with the parasite, but who have received treatment, unlike the sample used in the initial evaluation. It is therefore possible that our estimates reflect a more realistic evaluation of this test in the field.

Another issue with serum antibody ELISA tests in general is cross-reactivity with other trematodes [11]. The current ELISA showed no cross-reaction with *D. viviparus*, *N. helvetianus* and *O. ostertagi*, while cross-reaction with rumen flukes has not been reported [11]. Out of the 53 cattle with rumen flukes identified in the rumen and a negative liver necropsy result, 18 had positive sELISA results. While we cannot know whether those were animals with previous exposure to *F. hepatica* this may be an indication of cross-reactivity which can be further supported by Ibarra et al (1998) who reported cross-reactivity of an ES antigen ELISA first described by Arriaga de Morilla et al in 1989 with *Paramphistomum* spp. [45].

In our study most animals had a low fluke burden with a mean and median of 8.5 and 4 respectively. Firbrosis scores appeared to be related to burden, but it is important to note that our results explain the limitations of liver inspection by the MHS reflected in its imperfect sensitivity and specificity estimates. Presence of parasites in the liver did not always correspond to obvious fibrosis signs at inspection. Additionally, it is unclear whether “active” or “historic” is a useful classification as many of the livers classified as historic were found to harbour at least one fluke.

Even though knowing whether the infection is absent or present in an animal is highly important, one could argue that the level of infection present could also be important in the control of fasciolosis especially in cattle. As fasciolosis is a chronic disease in cattle causing mostly sub-clinical disease, it might be meaningful to farmers to know what the intensity of infection is and how that translates to production losses. This information might therefore be used to decide what treatment strategy if any they might decide to use [43]. Such an investigation was beyond the scope of this paper, but it is one definitely worth pursuing to investigate the use of available diagnostic tests in quantifying infection or level of production loss attributed to the infection for a more cost effective control of *F. hepatica* infection in cattle.

The present study has several strengths and limitations. We have used systematically chosen samples from naturally infected animals slaughtered at one of Scotland’s biggest abattoirs, therefore obtaining a sample more representative of the field situation than if experimentally infected animals where used [46]. Whilst we were not able to use simple random sampling due to logistics, we believe that this sampling method enabled us to represent animals arriving at the abattoir during the whole day. Five different tests were used in order to enable us to run a no gold standard analysis, avoiding the limitations of using an imperfect test as a gold standard. This approach certainly does not come without biases. In order to determine whether our proposed model could reclaim tests parameters using the sample size available, test results were simulated for three sub-populations of animals, representing the three sampling periods, under a range of plausible diagnostic test sensitivities and specificities. The model was run using this data and was able to recover pre-determined estimates of diagnostic test sensitivities/specificities and prevalence with reasonable precision for each sampling period. Furthermore, we checked for conditional dependence between tests and carried out appropriate MCMC diagnostics. Moreover, this is the first study to provide information on the appropriateness of available diagnostic tests during three different seasons, even though a first attempt at this was carried out by Charlier et al 2008 [39] using two sampling seasons, a much smaller dataset and a gold standard analysis.

Limitations of this study include the fact that we have not been able to account for differences in meat inspection results depending on which meat inspector carried out the inspection, as well as the fact that seasonal differences were described only during one year. If results of tests are dependent on the liver fluke life cycle, which in turn is heavily dependent on climatic factors, the appropriateness of diagnostic tests in each season might need to be tested over more years to confirm the differences or similarities described here. Additionally, we have assessed the assumption of conditional independence using pairwise dependency models. It is important to bear in mind that it is possible that more complicated dependencies might exist, which we were unable to account for. Nevertheless, due to the fact that the tests compared are looking for five different signals; flukes, fluke damage, eggs, faecal antigens and serum antibodies, it is unlikely that there are biologically likely common proxies of disease that might result in important covariance structures. This is supported by the absence of any considerable change in estimates using the 10 possible covariance pairs.

Overall, our study has provided a valuable insight in the performance of tests available for the diagnosis of *F. hepatica* infections in a population of cattle believed to be representative of the field situation. Knowing its limitations and being able to adjust for them, abattoir liver inspection, can be a valuable tool in monitoring and understanding the changing epidemiology of *F. hepatica* as well as evaluating farm health plans. Faecal egg counting has been shown to still be a valuable tool in the diagnosis of current *F. hepatica* infections, but one has to bear in mind that it is a weak test during periods where recent infections are expected. The copro-antigen ELISA is a comparable test that can be used throughout the year, with evidence to suggest that there is no cross-reaction with the increasingly prevalent rumen fluke parasite. This study also provided further evaluation of an in house ES antigen ELISA showing that while being a valuable test, its sensitivity and specificity estimates are lower in the field setting that previously reported. Liver fluke control is becoming increasing challenging in the UK, hence the qualitative and quantitative evaluation of available diagnostic tests, as well as development of better ones is an area where ongoing investigation is required.

## Supporting Information

S1 R Script Model specification. This script contains the code used for comparison of 5 diagnostic tests during 3 sampling periods. This model is an adaptation of the Hui & Walter [21] approach for the evaluation of diagnostic tests when a “gold standard” is not available.
(R) Click here for additional data file.

S1 Table Data used in Bayesian no gold standard model. Table shows the data used in the Bayesian no gold standard model. For each period there were 32 possible combinations of test results and the number of animals for each combination is shown here. A negative test result is shown by 0 and a positive test result is shown by 1.
(PDF)

S1 Fig Conditional Dependence. Figure shows the mean estimates of sensitivity and specificity of each test as estimated by the 10 different models accounting for covariance of one combination of two tests at a time. For example S1S2 is the model including covariance terms for tests 1 and 2 i.e. MHS liver inspection and liver necropsy and so on. The last estimate (NoCov) as well as the horizontal line on each plot shows the mean as estimated by the model with no covariance terms. Plots such as Se4 containing 3 lines show Se or Sp estimates that were allowed to vary between season. Based on this figure we concluded that even though estimates vary slightly above or below the lines, there are no major differences in estimates when accounting for covariance for the different combinations of tests and the model with no covariance terms. It was therefore justifiable to use a final model with no covariance terms.
(PDF)

S2 Fig Effect of priors. A comparison between prior and posterior distributions of model parameters is shown in these two figures. The top figure shows the mean and 95% Bayesian credibility intervals of each model parameter. Bayesian credibility intervals of posterior distributions are much narrower than the priors showing that results are heavily informed by the data. As described in the methodology the only informative prior was the one for the specificity of the liver necropsy, Sp_2_. This figure shows that even though the prior distribution is more informative the result is also informed by the data. Similarly the bottom figure shows the density plots of prior and posterior distributions and how prior distributions (except Sp_2_) are vague and posterior distributions are highly data driven being much narrower than the prior distributions.
(PDF)

S3 Fig Comparison of results of original model and model using non-informative priors. Figure shows the results of the original model and of a model using non-informative priors for comparison. The analysis was repeated using priors dbeta(1,1) for the Se and Sp of all tests to assess the effect of priors. There is no obvious alterations of results.
(PDF)

S4 Fig Correlation between model parameters. Figure shows the cross correlations between the parameters included in the model in each of the 3 MCMC chains. There is no obvious strong correlation between any combination of parameters.
(PDF)

## References

[1] Rojo-Vázquez F, Meana A, Valcárcel F, Martínez-Valladares M. Update on trematode infections in sheep. Veterinary Parasitology. 2012;189(1):15–38. 10.1016/j.vetpar.2012.03.029 22521973

[2] Andrews SJ. The life cycle of Fasciola hepatica In: Dalton JP, editor. Fasciolosis. Oxon: CABI publishing; 1999 p. 1–30.

[3] Taylor MA, Coop LR, Wall LR. Veterinary Parasitology. 3rd ed Oxford: Blackwell Publishing; 2007.

[4] European Commision, Directorate-General for Research and Innovation. A decade of EU-funded Animal Health. Luxembourg; 2012.

[5] Novobilský A, Sollenberg S, Höglund J. Distribution of *Fasciola hepatica* in Swedish dairy cattle and associations with pasture management factors. Geospatial health. 2015;9(2):293–300. 10.4081/gh.2015.351 25826310

[6] Graczyk TK, Bernard F. Development of *Fasciola hepatica* in the Intermediate Host In: Dalton JP, editor. Fasciolosis. Oxon: CABI publishing; 1999 p. 31–46.

[7] Ollerenshaw CB, Rowlands WT. A method of forecasting the incidence of fasciolosis in Anglesey. The Veterinary record. 1959;71(29):591–598.

[8] Mitchell G. Update on fasciolosis in cattle and sheep. In Practice. 2002;24(7):378–385. 10.1136/inpract.24.7.378

[9] Sargison ND, Scott PR. Diagnosis and economic consequences of triclabendazole resistance in *Fasciola hepatica* in a sheep flock in south-east Scotland. The Veterinary record. 2011;168(6):159 10.1136/vr.c5332 21493511

[10] Gordon DK, Zadoks RN, Stevenson H, Sargison ND, Skuce PJ. On farm evaluation of the coproantigen ELISA and coproantigen reduction test in Scottish sheep naturally infected with *Fasciola hepatica*. Veterinary parasitology. 2012;187(3-4):436–44. 10.1016/j.vetpar.2012.02.009 22421492

[11] Salimi-Bejestani MR, McGarry JW, Felstead S, Ortiz P, Akca A, Williams DJL. Development of an antibody-detection ELISA for *Fasciola hepatica* and its evaluation against a commercially available test. Research in veterinary science. 2005;78(2):177–81. 10.1016/j.rvsc.2004.08.005 15563926

[12] Mezo M, González-Warleta M, Carro C, Ubeira FM, Gonza M. An ultrasensitive capture ELISA for detection of *Fasciola hepatica* coproantigens in sheep and cattle using a new monoclonal antibody (MM3). Journal of Parasitology. 2004;90(4):845–852. 10.1645/GE-192R 15357080

[13] Flanagan AM, Edgar HWJ, Forster F, Gordon A, Hanna REB, McCoy M, et al Standardisation of a coproantigen reduction test (CRT) protocol for the diagnosis of resistance to triclabendazole in *Fasciola hepatica*. Veterinary parasitology. 2011;176(1):34–42. 10.1016/j.vetpar.2010.10.037 21093156

[14] Brockwell YM, Spithill TW, Anderson GR, Grillo V, Sangster NC. Comparative kinetics of serological and coproantigen ELISA and faecal egg count in cattle experimentally infected with *Fasciola hepatica* and following treatment with triclabendazole. Veterinary parasitology. 2013;196(3-4):417–426. 10.1016/j.vetpar.2013.04.012 23643623

[15] Rapsch C, Schweizer G, Grimm F, Kohler L, Bauer C, Deplazes P, et al Estimating the true prevalence of *Fasciola hepatica* in cattle slaughtered in Switzerland in the absence of an absolute diagnostic test. International journal for parasitology. 2006;36(10-11):1153–8. 10.1016/j.ijpara.2006.06.001 16843470

[16] Clery D, Torgerson P, Mulcahy G. Immune responses of chronically infected adult cattle to *Fasciola hepatica*. Veterinary parasitology. 1996;62(1-2):71–82. 10.1016/0304-4017(95)00858-6 8638395

[17] De Bont J, Claerebout E, Riveau G, Schacht AM, Smets K, Conder G, et al Failure of a recombinant *Schistosoma bovis*-derived glutathione S-transferase to protect cattle against experimental *Fasciola hepatica* infection. Veterinary Parasitology. 2003;113(2):135–44. 10.1016/S0304-4017(02)00450-8 12695038

[18] Happich FA, Boray JC. Quantitative diagnosis of chronic fasciolosis 1. Comparative Studies on Quantitative Faecal Examinations for Chronic. Australian Veterinary Journal. 1969;45(7):326–28. 10.1111/j.1751-0813.1969.tb05009.x 5817298

[19] Food Standards Agency. Post-Mortem, Health and Identification Marking. In: Manual for official controls; 2015.

[20] Sargison N, Francis E, Davison C, deC Bronsvoort BM, Handel I, Mazeri S. Observations on the biology, epidemiology and economic relevance of rumen flukes (*Paramphistomidae*) in cattle kept in a temperate environment. Veterinary Parasitology. 2016;219:7–16. 10.1016/j.vetpar.2016.01.010 26921033

[21] Hui S, Walter S. Estimating the error rates of diagnostic tests. Biometrics. 1980;36(1):167–71. 10.2307/2530508 7370371

[22] Branscum AJ, Gardner IA, Johnson WO. Estimation of diagnostic test sensitivity and specificity through Bayesian modeling. Preventive veterinary medicine. 2005;68(2-4):145–63. 10.1016/j.prevetmed.2004.12.005 15820113

[23] Toft N, Jørgensen E, Højsgaard S. Diagnosing diagnostic tests: Evaluating the assumptions underlying the estimation of sensitivity and specificity in the absence of a gold standard. Preventive Veterinary Medicine. 2005;68(1):19–33. 10.1016/j.prevetmed.2005.01.006 15795013

[24] deC Bronsvoort BM, Koterwas B, Land F, Handel IG, Tucker J, Morgan KL, et al Comparison of a flow assay for brucellosis antibodies with the reference cELISA test in West African *Bos indicus*. PloS ONE. 2009;4(4):e5221 10.1371/journal.pone.0005221 19381332 PMC2667634

[25] Vacek PM. The effect of conditional dependence on the evaluation of diagnostic tests. Biometrics. 1985;41(4):959–968. 10.2307/2530967 3830260

[26] Gardner IA, Stryhn H, Lind P, Collins MT. Conditional dependence between tests affects the diagnosis and surveillance of animal diseases. Preventive veterinary medicine. 2000;45(1-2):107–22. 10.1016/S0167-5877(00)00119-7 10802336

[27] Martyn P, Best N, Cowles K, Vines K. CODA: Convergence Diagnosis and Output Analysis for MCMC. R News. 2006;6(1):7–11.

[28] Chowaniec W, Darski J. Investigations on excretion time of liver fluke eggs after killing the parasite. Bulletin of the Veterinary Institute in Pulawy. 1970;14(3-4):108–110.

[29] Dendukuri N, Joseph L. Bayesian approaches to modeling the conditional dependence between multiple diagnostic tests. Biometrics. 2001;57(1):158–167. 10.1111/j.0006-341X.2001.00158.x 11252592

[30] Martyn P. JAGS: A program for analysis of Bayesian graphical models using Gibbs sampling. In: Proceedings of the 3rd International Workshop on Distributed Statistical Computing; 2003.

[31] R Core Development team. R: A Language and Environment for Statistical Computing; 2014. Available from: http://CRAN.R-project.org/package=rjags

[32] Martyn P. rjags: Bayesian graphical models using MCMC; 2014.

[33] Wei T. corrplot: Visualization of a correlation matrix; 2013. Available from: http://CRAN.R-project.org/package=corrplot

[34] Wickam H. ggplot2: elegant graphics for data analysis. Springer New York; 2009. Available from: http://had.co.nz/ggplot2/book

[35] Kahle D, Wickham H. ggmap: Spatial Visualization with ggplot2. The R Journal. 2013;5(1):144–161.

[36] Akobeng AK. Understanding diagnostic tests 1: sensitivity, specificity and predictive values. Acta paediatrica. 2007;96(3):338–341. 10.1111/j.1651-2227.2006.00180.x 17407452

[37] Price P, Bayes T. An Essay towards solving a problem in the doctrine of chances. by the Late Rev. Mr. Bayes, F. R. S. Communicated by Mr. Price, in a Letter to John Canton, A. M. F. R. S. Philosophical Transactions of the Royal Society. 1763;53:370–418. 10.1098/rstl.1763.0053

[38] Linn S. A New Conceptual Approach to Teaching the Interpretation of Clinical Tests. Journal of Statistics Education. 2004;12(3):1–9.

[39] Charlier J, De Meulemeester L, Claerebout E, Williams DJL, Vercruysse J. Qualitative and quantitative evaluation of coprological and serological techniques for the diagnosis of fasciolosis in cattle. Veterinary parasitology. 2008;153(1-2):44–51. 10.1016/j.vetpar.2008.01.035 18329811

[40] Palmer D, Lyon J, Palmer M, Forshaw D. Evaluation of a copro-antigen ELISA to detect *Fasciola hepatica* infection in sheep, cattle and horses. Australian Veterinary Journal. 2014;92(9):357–361. 10.1111/avj.12224 25156056

[41] Gordon DK, Roberts LCP, Lean N, Zadoks RN, Sargison ND, Skuce PJ. Identification of the rumen fluke, *Calicophoron daubneyi*, in GB livestock: possible implications for liver fluke diagnosis. Veterinary parasitology. 2013;195(1-2):65–71. 10.1016/j.vetpar.2013.01.014 23411375

[42] Kajugu PE, Hanna REB, Edgar HW, Forster FI, Malone FE, Brennan GP, et al Specificity of a coproantigen ELISA test for fasciolosis: lack of cross-reactivity with *Paramphistomum cervi* and *Taenia hydatigena*. Veterinary Record. 2012;171(20):502 10.1136/vr.101041 23077134

[43] Charlier J, Vercruysse J, Morgan E, van Dijk J, Williams DJL. Recent advances in the diagnosis, impact on production and prediction of *Fasciola hepatica* in cattle. Parasitology. 2014;141(3):326–35. 10.1017/S0031182013001662 24229764

[44] Alvarez Rojas CA, Jex AR, Gasser RB. Chapter Two Techniques for the Diagnosis of *Fasciola* Infections in Animals: Room for Improvement. Advances in Parasitology. 2014;85:65–107. 10.1016/B978-0-12-800182-0.00002-7 24928180

[45] Arriaga de Morilla C, Paniagua R, Ruiz-Navarrete A, Bautista C, Morilla A. Comparison of dot enzyme-linked immunosorbent assay (Dot-ELISA), passive haemagglutination test (PHT) and thin layer immunoassay (TIA) in the diagnosis of natural or experimental *Fasciola hepatica* infections in sheep. Veterinary parasitology. 1989;30(3):197–203. 10.1016/0304-4017(89)90015-0 2705286

[46] Salimi-Bejestani MR, Cripps P, Williams DJL. Evaluation of an ELISA to assess the intensity of *Fasciola hepatica* infection in cattle. The Veterinary record. 2008;162(4):109–111. 10.1136/vr.162.4.109 18223266

