# Supplementary material for: Evaluation of the Performance of Five Diagnostic Tests for Fasciola hepatica Infection in Naturally Infected Cattle Using a Bayesian No Gold Standard Approach
Source: PLoS One. 2016 Aug 26;11(8):e0161621. doi: 10.1371/journal.pone.0161621 (PMC5001639; doi:10.1371/journal.pone.0161621)
Supplement: S1 Table — Table shows the data used in the Bayesian no gold standard model. For each period there were 32 possible combinations of test results and the number of animals for each combination is shown here. A negative test result is shown by 0 and a positive test result is shown by 1. (PDF) [file pone.0161621.s002.pdf]

Combination	Test result (0=negative, 1=positive)					Number of animals		
	cELISA	FEC	sELISA	MHS	Necropsy	Period A	Period B	Period C
1	1	1	1	1	1	32	31	15
2	1	1	1	0	1	9	10	6
3	1	1	1	1	0	0	0	0
4	1	1	1	0	0	0	0	0
5	0	1	1	1	1	4	4	0
6	0	1	1	0	1	1	2	1
7	0	1	1	1	0	0	0	0
8	0	1	1	0	0	0	0	0
9	1	0	1	1	1	4	7	9
10	1	0	1	0	1	2	4	2
11	1	0	1	1	0	0	0	0
12	1	0	1	0	0	1	0	0
13	0	0	1	1	1	4	1	4
14	0	0	1	0	1	1	1	2
15	0	0	1	1	0	7	3	14
16	0	0	1	0	0	8	11	23
17	1	1	0	1	1	5	0	1
18	1	1	0	0	1	9	1	3
19	1	1	0	1	0	0	0	0
20	1	1	0	0	0	0	0	1
21	0	1	0	1	1	4	0	0
22	0	1	0	0	1	0	1	0
23	0	1	0	1	0	0	0	1
24	0	1	0	0	0	1	1	0
25	1	0	0	1	1	0	1	1
26	1	0	0	0	1	2	0	3
27	1	0	0	1	0	0	0	0
28	1	0	0	0	0	0	1	0
29	0	0	0	1	1	2	1	0
30	0	0	0	0	1	1	4	1
31	0	0	0	1	0	4	12	7
32	0	0	0	0	0	106	108	114
