# Supplementary material for: Evaluation of the Performance of Five Diagnostic Tests for Fasciola hepatica Infection in Naturally Infected Cattle Using a Bayesian No Gold Standard Approach
Source: PLoS One. 2016 Aug 26;11(8):e0161621. doi: 10.1371/journal.pone.0161621 (PMC5001639; doi:10.1371/journal.pone.0161621)
Supplement: S1 Fig — Figure shows the mean estimates of sensitivity and specificity of each test as estimated by the 10 different models accounting for covariance of one combination of two tests at a time. For example S1S2 is the model including covariance terms for tests 1 and 2 i.e. MHS liver inspection and liver necropsy and so on. The last estimate (NoCov) as well as the horizontal line on each plot shows the mean as estimated by the model with no covariance terms. Plots such as Se4 containing 3 lines show Se or Sp estimates that were allowed to vary between season. Based on this figure we concluded that even though estimates vary slightly above or below the lines, there are no major differences in estimates when accounting for covariance for the different combinations of tests and the model with no covariance terms. It was therefore justifiable to use a final model with no covariance terms. (PDF) [file pone.0161621.s003.pdf]

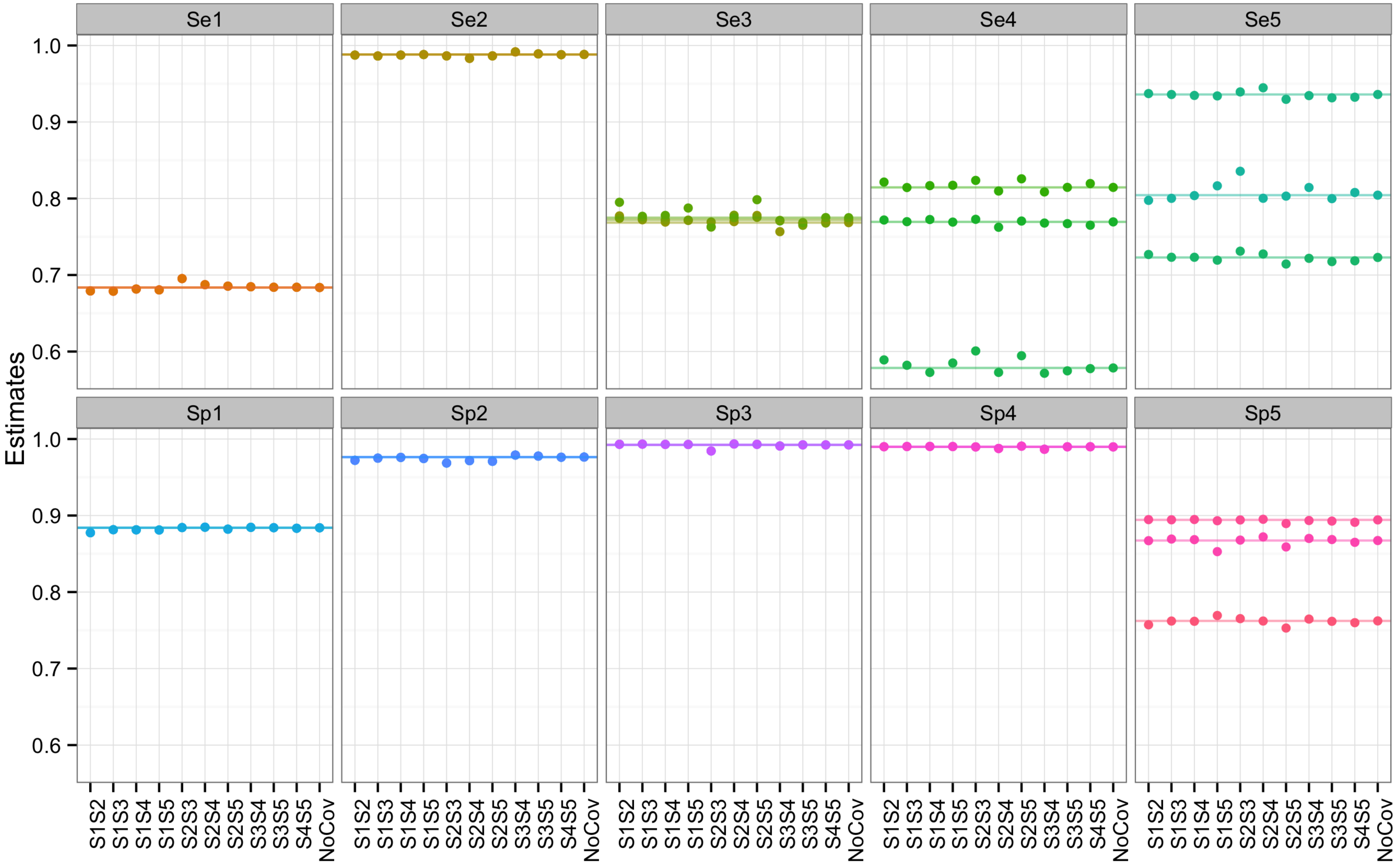

Mean estimates of sensitivity (Se) and specificity (Sp) for each test accounting for covariance for one combination of tests at a time. NoCov and straight lines show the estimate when no covariance terms are included (1 - MHS, 2 - Necropsy, 3 - cELISA, 4 - FEC, 5 - sELISA).
