# Supplementary material for: Evaluation of the Performance of Five Diagnostic Tests for Fasciola hepatica Infection in Naturally Infected Cattle Using a Bayesian No Gold Standard Approach
Source: PLoS One. 2016 Aug 26;11(8):e0161621. doi: 10.1371/journal.pone.0161621 (PMC5001639; doi:10.1371/journal.pone.0161621)
Supplement: S2 Fig — A comparison between prior and posterior distributions of model parameters is shown in these two figures. The top figure shows the mean and 95% Bayesian credibility intervals of each model parameter. Bayesian credibility intervals of posterior distributions are much narrower than the priors showing that results are heavily informed by the data. As described in the methodology the only informative prior was the one for the specificity of the liver necropsy, Sp2. This figure shows that even though the prior distribution is more informative the result is also informed by the data. Similarly the bottom figure shows the density plots of prior and posterior distributions and how prior distributions (except Sp2) are vague and posterior distributions are highly data driven being much narrower than the prior distributions. (PDF) [file pone.0161621.s004.pdf]

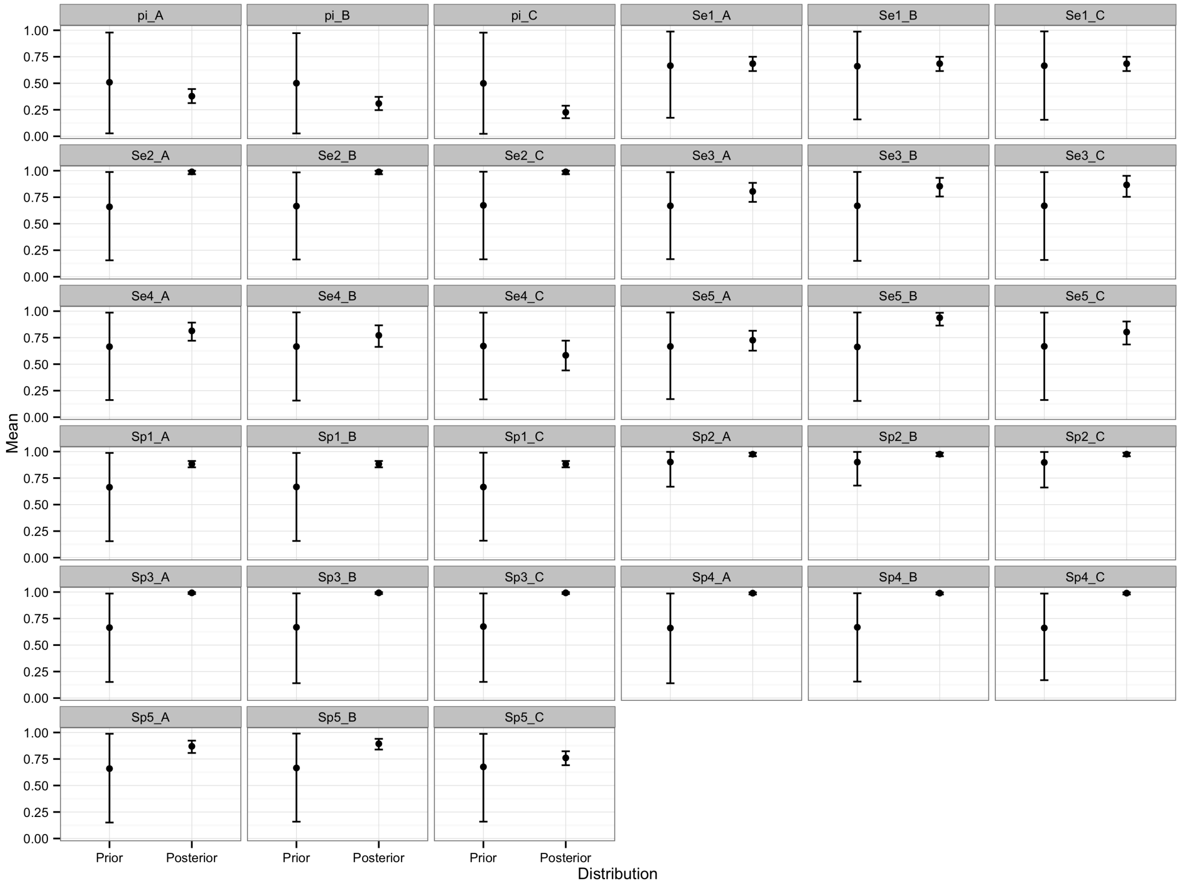

Comparison between prior and posterior distributions of model parameters by mean and 95% Bayesian credibility Intervals (pi - prevalence, 1 - MHS, 2 - Necropsy, 3 - cELISA, 4 - FEC, 5 - sELISA, A - summer 2013, B - winter 2014, C - autumn 2014).

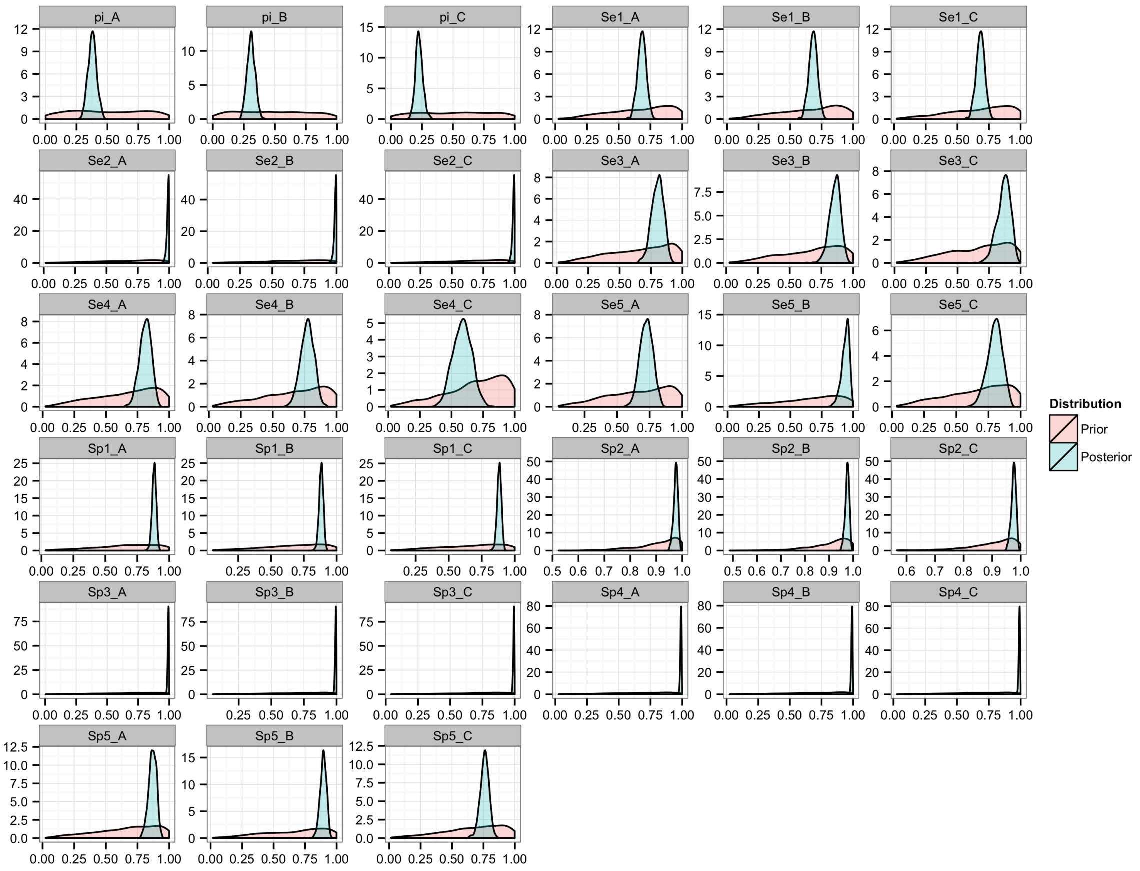

Comparison between prior and posterior distributions of model parameters using density plots (pi - prevalence, 1 - MHS, 2 - Necropsy, 3 - cELISA, 4 - FEC, 5 - sELISA, A - summer 2013, B - winter 2014, C - autumn 2014).
