# Supplementary figures and images for: Evaluation of the Performance of Five Diagnostic Tests for Fasciola hepatica Infection in Naturally Infected Cattle Using a Bayesian No Gold Standard Approach
Source: PLoS One. 2016 Aug 26;11(8):e0161621. doi: 10.1371/journal.pone.0161621 (PMC5001639; doi:10.1371/journal.pone.0161621)

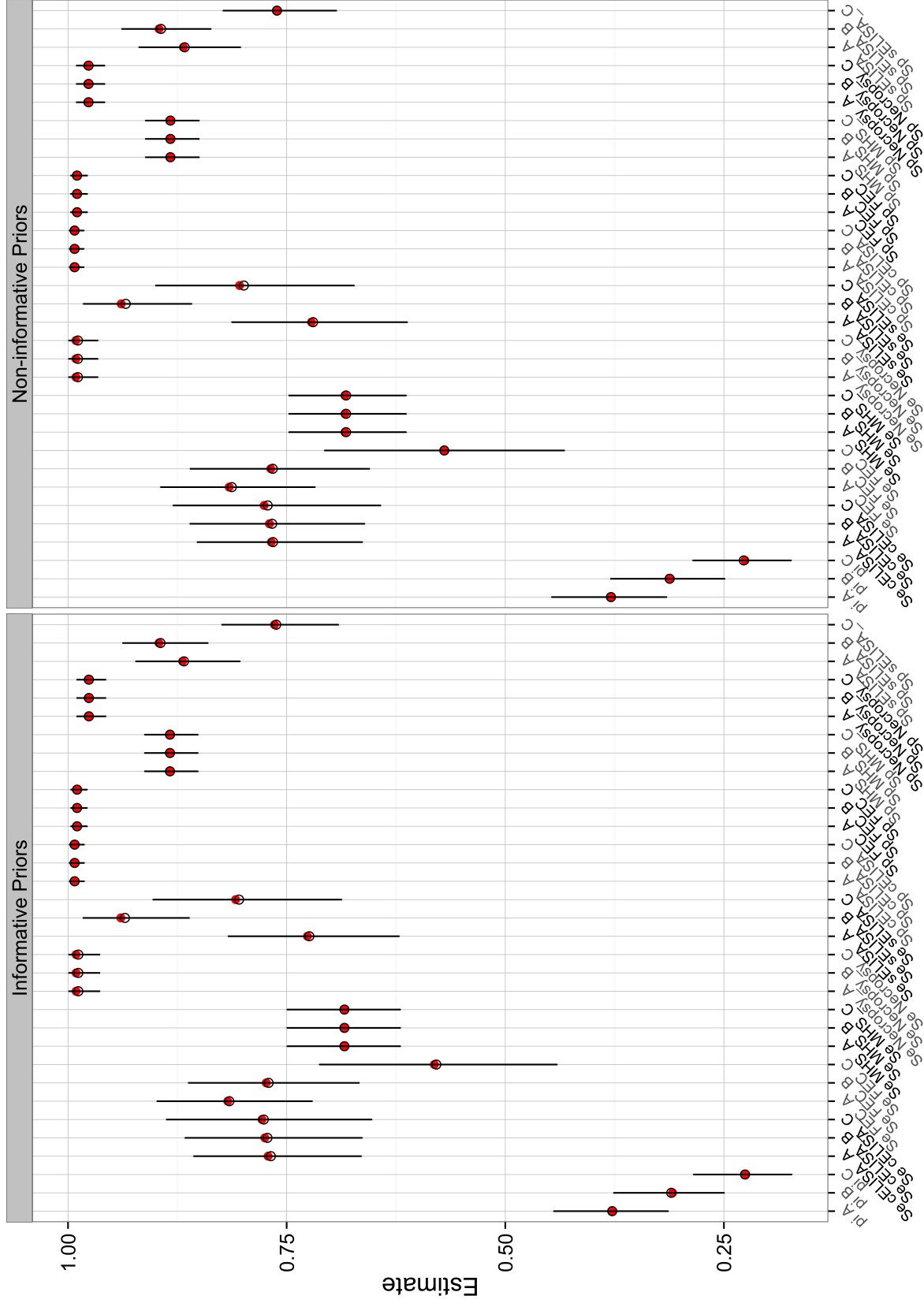

### Comparison between results of the original model and of a model using non-informative priors.

Supplement: S3 Fig — Figure shows the results of the original model and of a model using non-informative priors for comparison. The analysis was repeated using priors dbeta(1,1) for the Se and Sp of all tests to assess the effect of priors. There is no obvious alterations of results. (PDF) [file pone.0161621.s005.pdf]
