# Supplementary material for: Evaluation of the Performance of Five Diagnostic Tests for Fasciola hepatica Infection in Naturally Infected Cattle Using a Bayesian No Gold Standard Approach
Source: PLoS One. 2016 Aug 26;11(8):e0161621. doi: 10.1371/journal.pone.0161621 (PMC5001639; doi:10.1371/journal.pone.0161621)
Supplement: S4 Fig — Figure shows the cross correlations between the parameters included in the model in each of the 3 MCMC chains. There is no obvious strong correlation between any combination of parameters. (PDF) [file pone.0161621.s006.pdf]

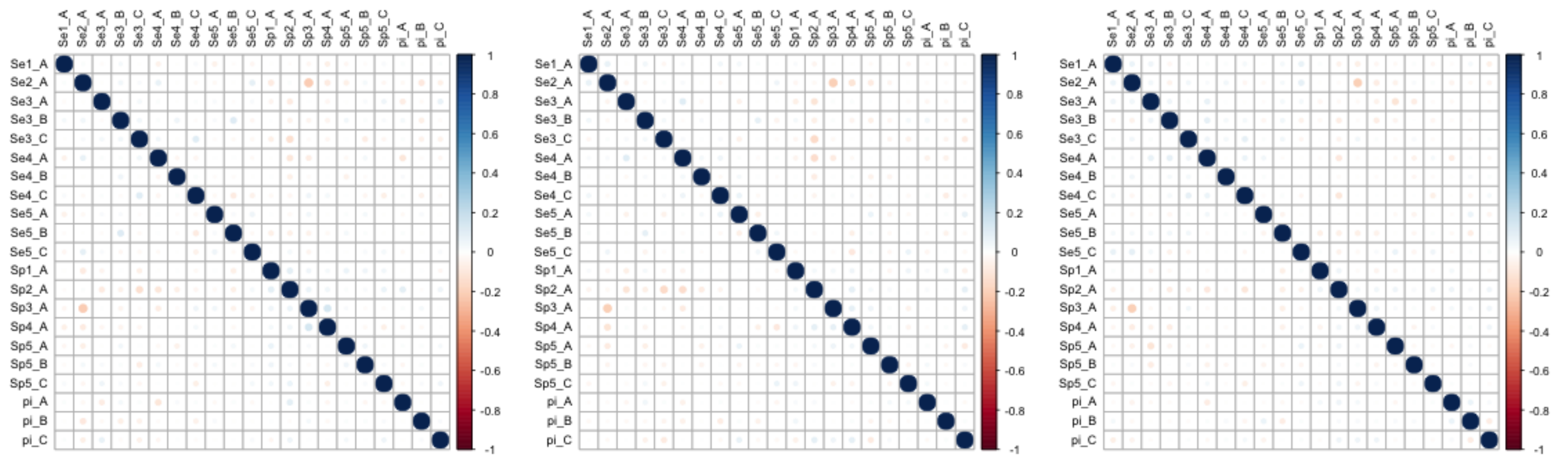

Correlation matrix of the variables included in the model for chains 1 to 3 (left to right). Se - sensitivity, Sp - specificity, pi - prevalence, 1 - MHS, 2 - Necropsy, 3 - cELISA, 4 - FEC, 5 - sELISA, A - summer 2013, B - winter 2014, C - autumn 2014.
